# Spontaneous Macular Hole Closure after Valsalva Retinopathy and Nd:YAG Laser Treatment

**DOI:** 10.1155/2018/1782847

**Published:** 2018-08-23

**Authors:** Elcin Suren, Melih Akidan, Muhammet Kazim Erol, Devrim Toslak

**Affiliations:** ^1^Antalya Education and Research Hospital, Ophthalmology Department, Antalya, Turkey; ^2^Kepez State Hospital, Ophthalmology Department, Antalya, Turkey

## Abstract

**Purpose:**

The purpose of this report is to present a case who had spontaneous macular hole closure after Nd:YAG laser membranotomy applied to premacular haemorrhage associated with Valsalva retinopathy.

**Methods:**

Case report.

**Results:**

A 19-year-old young male patient presented to our clinic with sudden vision loss in his right eye, which had occurred 2 weeks before, following push-up and sit-up exercise. The patient was found to have premacular haemorrhage associated with Valsalva retinopathy. Nd:YAG laser membranotomy was performed. During his follow-up at week 1, full-thickness MH was observed and he was put under observation. At month 6, his vision acuity improved, laser coagulation sites in the fundus disappeared, and macular hole closed spontaneously.

**Conclusion:**

Macular hole that develops after Nd:YAG laser treatment of Valsalva retinopathy may spontaneously be closed like in our case. However, there is a need for further research to understand the mechanism of closure.

## 1. Introduction

Valsalva retinopathy occurs when the superficial retinal capillaries are ruptured as a result of elevated intraocular venous pressure that occurs following an abrupt increase in intrathoracic or intra-abdominal pressure and usually leads to preretinal haemorrhage in the macula [1]. The premacular haemorrhage is anatomically located either under the internal limiting membrane (ILM) or under the posterior hyaloid or both [2].

Observation, neodymium-doped yttrium aluminium garnet (Nd:YAG) laser membranotomy, and vitrectomy are among the management options. Complications such as macular hole, retinal detachment, epiretinal membrane formation, and persistent premacular cavity may develop after Nd:YAG laser treatment [3].

Spontaneous closure of macular hole may occur in trauma-related cases, postvitrectomy, or may be idiopathic [4]. Spontaneous resolution may also be observed albeit rarely after Valsalva retinopathy.

We present a patient whose macular hole that developed after Nd:YAG laser membranotomy performed to treat premacular haemorrhage associated with Valsalva retinopathy was closed spontaneously during his follow-up. To the best of our knowledge, there has been no case reported to have complete spontaneous macular hole resolution of Valsalva retinopathy after laser treatment.

## 2. Case Report

**Figure 1:**
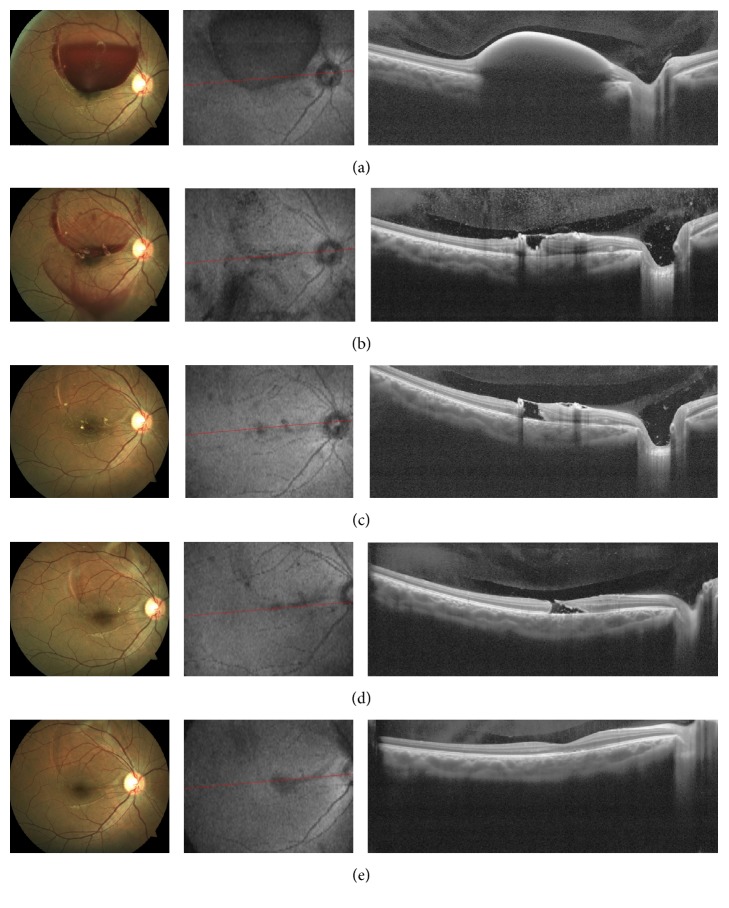
Valsalva retinopathy in the right eye, colour fundus photographs, and Enhanced Deep Image (EDI) optical coherence tomography (OCT) cross-sections. (a) Before Nd:YAG laser membranotomy. (b) 1st hour after the procedure. (c) At week 1, laser spot sites are distinguished in the fundus, full-thickness macular hole in the EDI-OCT. (d) At month 3, full-thickness macular hole persisted. (e) At month 6, macular hole closed spontaneously.

A 19-year-old young male patient presented to our clinic with sudden vision loss in his right eye 2 weeks after push-up and sit-up exercise. He was admitted to another centre before; he was told that he had haemorrhage in his eye and was advised only follow-up. He had no history of trauma, ocular, or systemic disease. His examination showed that his best corrected visual acuity (BCVA) was counting fingers from 1 meter distance in the right eye, 20/20 in the left eye. Anterior segment examination and intraocular pressure findings were unremarkable. Fundus examination of his right eye showed a well-circumscribed, elevated, and around 4 disc diameter (DD) premacular haemorrhage that extended from the upper vascular arcuate to the fovea. Colour fundus photography and fundus fluorescein angiography were performed. Spectral-domain optical coherence tomography (SD-OCT) revealed dome-shaped elevated lesion with a hyperreflective surface and hyporeflective area underneath (Figure 1(a)). It was suspected to be the combination of subhyaloid haemorrhage with wider localization and sub-ILM haemorrhage localized only in the fovea. Complete blood counts and biochemical and clotting parameter were ordered for Valsalva retinopathy and other causes of premacular haemorrhage for the differential diagnosis. Internal diseases clinic was consulted. After all the other evaluations, he was diagnosed with Valsalva retinopathy. Treatment options were explained to the patient. He preferred laser treatment.

Nd:YAG laser (VISULAS YAG III, Carl Zeiss Meditec AG, Jena, Germany) and Ocular Abraham Capsulotomy YAG Laser Lens were performed with a posterior approach. The initial energy of laser exposures was 1.9 mJ and then it was gradually increased until it was observed that a rapid stream of blood was trapped into the vitreous cavity. Laser energy was carefully shot onto the anterior surface and inferior margin of the haemorrhage away from the fovea and two openings were made.

At the first hour after the procedure, haemorrhage was resolved considerably (Figure 1(b)). During his follow-up 2 days later, the laser shot sites could be clearly observed and there were only peripherally localized sub-ILM haemorrhage sites. Although his fundus findings were observed to be resolved totally during his control at week 1, BCVA was counting fingers from 3 meter. SD-OCT revealed full-thickness macular hole (Figure 1(c)). He did not have a marked vision acuity at month 3 and his macular hole findings persisted (Figure 1(d)). At month 6, BCVA was 20/50, and his fundus examination showed that laser coagulation sites disappeared. SD-OCT revealed the macular hole was spontaneously closed and the normal foveal contour was reformed (Figure 1(e)). At month 12, the fundus examination and SD-OCT findings were stable, the final visual acuity was 20/32.

## 3. Discussion

Valsalva retinopathy is associated with a number of clinical settings among young adults such as intense aerobic exercise, heavy lifting, straining on the toilet, vomiting, and coughing [5]. Premacular haemorrhage may develop secondary to Valsalva retinopathy while sub-ILM haemorrhage is more common than subhyaloid haemorrhage [6]. In our case, both fundus examination and SD-OCT revealed that a large subhyaloid haemorrhage was combined with a small sub-ILM haemorrhage localized in the fovea.

In most of the cases, haemorrhage may be self-limited, for which conservative treatment is widely used. Spontaneous absorption, however, is very slow and sometimes it takes several months for even a small premacular haemorrhage to resolve. In such a case, there is a likelihood that haemoglobin and iron have a toxic effect on retina [7]. Nd: YAG laser membranotomy is a widely used noninvasive method that usually improves vision acuity rapidly especially in cases of haemorrhage above 3 DD [8]. As regards complications, there is a small risk of epiretinal membrane, premacular cavity formation, and ILM thickening [9]. Another treatment options is vitrectomy whereas it is usually recommended for cases with dense haemorrhage and insufficient reabsorption that do not respond to the other treatments due to its potential complications and costs [10]. Although successful results were attained with ranibizumab and intravitreal injections of tissue plasminogen activator (tPA) after pneumopexy, there is still a need for larger studies [11, 12].

Regression in premacular haemorrhage associated with Valsalva retinopathy was achieved while macular hole may develop. Xie et al. observed a case whose haemorrhage regressed but then lamellar macular hole developed after 17 months [13]. Haemorrhage breaking through the neurosensory layers, tangential traction on the fovea by thickened or detached ILM, and toxicity of the long-lasting blood are suggested as possible mechanisms. Kim YK et al. performed second vitrectomy by leaving gas after macular hole developed in vitrectomized cases whose preretinal membranes and ILM were stripped before, and they achieved resolution [14]. They thought that it was associated with iatrogenic or thick preretinal membrane's tangential tractional force over the fovea and absorption of sub-ILM deposits. Bypareddy et al. performed Nd:YAG laser similar to our case and reported that macular hole developed which was associated with direct impact of the laser beam or the disruption of a detached ILM having some adhesions to the underlying retina [15]. In fact, larger studies have demonstrated higher success rates with Nd:YAG laser, good visualisation, and low complication rates. Durukan et al. did not find any complication after laser treatment in their study including 16 patients [16]. Ulbig et al. determined rhegmatogenous retinal detachment in 1 myopic patient and macular hole in 1 patient in their series of 21 patients who received laser treatment [17]. The common recommendation is that low energy level should be used and membranotomy procedure should be performed away from the fovea as much as possible.

In our case, after the total resolution of haemorrhage, an examination of the areas where laser shots were applied revealed that they were close to the fovea and the contact site with retina was slightly wide. At month 6, however, recovery of the laser sites without any scar was an indication of the fact that energy level we applied was not high enough to affect full thickness. We think that macular hole developed to the similar reasons mentioned above and traction effect decreased in time although a hole with wide diameter developed initially, which allowed glial cell proliferation to bridge the gap between the edges; thus this led to spontaneous closure.

In conclusion, laser shot and the energy level applied are the important factors to minimize the complications after Nd:YAG laser treatment of premacular haemorrhage secondary to Valsalva retinopathy. Macular hole develops rarely after Nd:YAG laser treatment, while it may be spontaneously closed like in our case. There is a need for further research to understand the mechanism of closure. Furthermore, it should be remembered that spontaneous resolution may occur before early surgery or alternative treatments of macular hole.
